# Nutritional Risk Factors for Age-Related Macular Degeneration

**DOI:** 10.1155/2014/413150

**Published:** 2014-07-03

**Authors:** Lebriz Ersoy, Tina Ristau, Yara T. Lechanteur, Moritz Hahn, Carel B. Hoyng, Bernd Kirchhof, Anneke I. den Hollander, Sascha Fauser

**Affiliations:** ^1^Department of Ophthalmology, University Hospital of Cologne, Kerpener Straße 62, 50924 Cologne, Germany; ^2^Department of Ophthalmology, Radboud University Nijmegen Medical Center, Geert Grooteplein-Zuid 10, 6525 GA Nijmegen, The Netherlands; ^3^Institute of Medical Statistics, Informatics and Epidemiology, University of Cologne, Kerpener Straße 62, 50924 Cologne, Germany

## Abstract

*Purpose*. To evaluate the role of nutritional factors, serum lipids, and lipoproteins in late age-related macular degeneration (late AMD). *Methods*. Intake of red meat, fruit, fish, vegetables, and alcohol, smoking status, and body mass index (BMI) were ascertained questionnaire-based in 1147 late AMD cases and 1773 controls from the European Genetic Database. Serum levels of lipids and lipoproteins were determined. The relationship between nutritional factors and late AMD was assessed using logistic regression. Based on multivariate analysis, area-under-the-curve (AUC) was calculated by receiver-operating-characteristics (ROC).* Results*. In a multivariate analysis, besides age and smoking, obesity (odds ratio (OR): 1.44, *P* = 0.014) and red meat intake (daily: OR: 2.34, *P* = 8.22 × 10^−6^; 2–6x/week: OR: 1.67, *P* = 7.98 × 10^−5^) were identified as risk factors for developing late AMD. Fruit intake showed a protective effect (daily: OR: 0.52, *P* = 0.005; 2–6x/week: OR: 0.58, *P* = 0.035). Serum lipid and lipoprotein levels showed no significant association with late AMD. ROC for nutritional factors, smoking, age, and BMI revealed an AUC of 0.781.* Conclusion*. Red meat intake and obesity were independently associated with increased risk for late AMD, whereas fruit intake was protective. A better understanding of nutritional risk factors is necessary for the prevention of AMD.

## 1. Introduction

Age-related macular degeneration (AMD) is the leading cause of severe visual impairment in developed countries. As the proportion of elderly is growing rapidly, AMD has become a major global public health concern [1]. AMD can be classified into a neovascular (wet) and an atrophic (dry) form. Although therapy with intraocular drugs inhibiting vascular endothelial growth factor has had a substantial effect on reducing visual impairment for wet AMD [2], no effective therapy is available for dry AMD. Therefore, identification of modifiable risk factors is a key approach to reduce the burden of the disease.

The age-related eye disease study (AREDS) investigated the role of dietary supplements in the progression of AMD. A formulation of vitamin C, vitamin E, beta-carotene, and zinc showed a 34% risk reduction in progression from intermediate to late stages over 6 years follow-up [3]. Current data about the beneficial role of increased dietary intake of carotenoids and antioxidants on AMD are not consistent [4–7].

The epidemiological evidence regarding the relationship between dietary fat intake and AMD is also controversial [6]. Fatty fish intake has been linked to high levels of polysaturated fatty acids and to a decreased risk of AMD [8, 9], whereas other studies reported increased total fat intake as a risk factor for AMD [10]. Some studies have found no significant association between dietary fat intake and AMD, after adjustment for confounders [11]. No beneficial effects of additional intake of long chain omega 3 polyunsaturated fatty acids were reported in the interventional AREDS2 [7].

These inconsistent data might be explained by the complex interaction between nutritional components and other environmental factors. Thus, rather than focusing on one particular component of food, we decided to examine AMD risk in relation to various nutritional factors.

## 2. Material and Methods

### 2.1. Study Population

Study participants were recruited from the multicenter European Genetic Database (EUGENDA, http://www.eugenda.org/) from Nijmegen, the Netherlands, and Cologne, Germany, for the clinical and molecular analysis of AMD. This study was performed in accordance with the tenets of the Declaration of Helsinki (1983 revision) and the Medical Research Involving Human Subjects Act (WMO). Written informed consent was obtained from all participants and the research protocols were approved by the local ethics committee of the University Hospital of Cologne.

Grading of retinal images, including stereo fundus photographs, fluorescein angiograms, and spectral domain ocular coherence tomograms, was performed for AMD staging. AMD was classified by the presence of more than 10 small drusen (<63 *μ*m) and pigmentary changes or presence of intermediate (64–124 *μ*m) or large drusen (≥125 *μ*m). Late AMD was defined as either AMD with subfoveal geographic atrophy (>175 *μ*m) or choroidal neovascularization. The more severely affected eye was used for disease classification. Patients with poor image quality, images of only one eye, and other diseases that may impede the diagnosis of AMD were not included in this study. Subjects with no AMD served as controls.

Demographic data and dietary intake of different food groups were assessed by a standardized interviewer-assisted questionnaire. Specifically, Specifically, participants were asked about their consumption of the following aliments: red meat (a portion size of 100–120 g), fish (a portion size of 100–120 g), fruits, and vegetables in the present time. Subjects could choose between four answers: daily intake, a few times a week, once a week, less than once a week.

Additionally, data about smoking (never/ever) and alcohol use (regular consumption of alkoholic drinks, e.g., 250 mL beer or 125 mL wine, at least weekly versus irregular or no alkohol intake) were evaluated.

Subjects were not excluded for missing data or odd answers to dietary questions. The group of no answers is presented in Table 1.

BMI was calculated based on self-reported weight and height and was divided into three groups (<25, 25–29.99, ≥30). Serum levels of apolipoprotein B (apo B), apolipoprotein A2 (apo A2), lipoprotein a (Lp(a)), total cholesterol, triglycerides, and HDL-cholesterol (HDLC) were measured with standard procedures by a clinical chemistry laboratory (Architect Analyzer; Abbott Diagnostics Hoofddorp, The Netherlands).

### 2.2. Statistical Analysis

All calculations were carried out using SPSS software version 21.0 (IBM Software and Systems, Armonk, NY, USA). Odds ratios (OR) and 95% confidence intervals (CI) were calculated by univariate logistic regression analysis separately for each food group, BMI, and alcohol use and adjusted for age, gender (female/male), and smoking (never/ever) before being combined in a multivariate logistic regression model.

Based on the multivariate logistic regression, an estimate for the probability of late AMD was calculated with the equation *P* = exp⁡(log⁡*it*(*P*))/(1 + exp⁡(log⁡*it*(*P*))) and used to determine the receiver-operating-characteristics (ROC) curve.

Differences between late AMD and control subjects in mean serum lipid and lipoprotein levels were tested using Mann-Whitney *U* test. *P* values ≤0.05 were considered statistically significant.

## 3. Results

From a total of 2920 participants included in the study, 1773 (60.72%) were healthy controls and 1147 (39.28%) showed late AMD.

Demographics and clinical parameters of the study population are shown in Table 1.

The associations of late AMD for each risk factor by univariate binary logistic regression analysis adjusted for age, gender, and smoking are summarized in Table 2. BMI and red meat consumption was significantly associated with increased risk for late AMD, while fruit intake seemed to play a protective role.

### 3.1. Multivariate Analysis

Based on multiple regression analysis on food groups (red meat, fish, and fruit), alcohol consumption, BMI, smoking, age, and gender, a model for late AMD was generated (Table 3). Due to the small number of cases in some subgroups (<1%) vegetable intake was not included in the multivariate analysis.

Besides age and a positive history of smoking, red meat intake (daily intake OR: 2.34, *P* = 8.22 × 10^−6^; 2–6x/week OR: 1.67, *P* = 7.98 × 10^−5^) and obesity (BMI ≥ 30, OR: 1.44, *P* = 0.014) were identified as dose-dependent risk factors for the development of late AMD, whereas regular fruit intake (daily intake OR: 0.52, *P* = 0.005; 2–6x/week OR: 0.58, *P* = 0.035) was a protective factor. No significant associations were found for fish and alcohol. The area under the curve (AUC) of the receiver-operating curve for the model was 0.781 (*P* = 1.00 × 10^−13^).

### 3.2. Lipids and Lipoproteins

No significant difference was observed in serum levels of apo B (*P* = 0.08), apo A2 (*P* = 0.12), Lp(a) (*P* = 0.71), total cholesterol (*P* = 0.43), HDLC (*P* = 0.52), and triglycerides (*P* = 0.47) between late AMD patients and controls.

The consumption of different food groups was not associated with differences in serum levels of apo B, apo A2, Lp(a), cholesterol, HDLC, and triglycerides. Among subjects with regular alcohol consumption, median level of triglycerides was lower (1.63 ± 0.89 versus 1.74 ± 1.02 mmol/L, *P* = 0.002) and median HDLC levels were slightly elevated (1.44 ± 0.37 versus 1.42 ± 0.35, *P* = 0.04) compared to persons without regular alcohol use.

## 4. Discussion

This study investigated the role of nutritional factors in late AMD. Red meat intake was identified as a risk factor and fruit intake as a protective factor, both in a dose-dependent manner. A risk model, based on nutrition, alcohol intake, and BMI adjusted for age, gender, and smoking, revealed a good discrimination between no AMD and late AMD, emphasizing the importance of modifiable risk factors in late AMD (AUC = 0.781). No significant association was found between fish or vegetable intake and late AMD in all groups.

Several studies previously suggested red meat intake as a risk factor for AMD, mostly by evaluation of dietary fat intake [6, 10, 12]. Red meat is also considered to be a major dietary risk factor for atherosclerosis. Although the focus has been on high cholesterol and saturated fatty acids content of red meat [13], recent studies point out that other ingredients, such as sodium, nitrites, nitrous compounds, as well as other preservatives present in processed meat, may be responsible for the cardiovascular risk [14]. Both red meat and smoking are associated with preformed N-nitroso compounds (NOCs) [15] and advanced glycated end products (AGEs) [16]. NOCs have been reported to damage the retina and AGEs stimulate vascular endothelial growth factors in retinal pigment epithelium cells [17] suggesting a possible role in the development of choroidal neovascularization.

Red meat also contains some presumably protective nutrients such as zinc. However, dietary intake of zinc from red meat but not from other sources was associated with a greater risk for cardiovascular disease, probably due to an interaction between heme iron and zinc metabolism [18]. This may also be an explanation for the AMD risk associated with red meat consumption. Our finding is in line with results from a cohort study which also found an association of red meat with late AMD [19].

The current study detected a protective effect of fruit consumption against late AMD (daily intake OR: 0.52). Fruits and vegetables contain various potentially protective components: vitamins, lutein, and zeaxanthin [20], while the use of the original AREDS formulation containing vitamin C, E, beta-carotene, and zinc showed a risk reduction of 34% in progression of AMD within 6 years [3]; retrospective review of the literature showed inconclusive data for the additional intake of carotenoids, vitamins, and other antioxidants [4–6]. It is also possible that other components of fruit are protective in AMD.

Consumption of regular fatty fish has been associated with increased serum polyunsaturated fatty acids such as docosahexaenoic acid (DHA) and eicosapentaenoic acid (EPA), and high levels of fish intake have been linked to a decreased risk of AMD [6, 8, 9]. In a recent study, a high red blood cell membrane EPA and DHA index (omega-3 index) was protective for neovascular AMD [21]. Our current study did not detect an association between regular fish intake and late AMD but this might be also due to the low fish intake frequencies in our data set (only 13 subjects reported a daily intake). However, our results are in accordance with the results of the ARED2 study where addition of DHA and EPA to the original AREDS formulation showed no protective role for AMD progression [7].

Moderate alcohol consumption is a protective factor in cardiovascular disease [22] and has been suggested to be protective for AMD [23], while heavy alcohol consumption has been associated with an increased risk of AMD [24]. The current study results support the beneficial effect of regular alcohol consumption on cardiovascular disease (lower mean BMI, slightly elevated HDLC, and decreased total triglyceride levels). However no association between alcohol consumption and late AMD was detected in our cohort.

Previous genetic studies have identified significant associations between AMD and polymorphisms in genes of the lipid metabolism [25]. Currently it is not certain how these polymorphisms influence the development of AMD, and the causal effect of serum lipids and lipoproteins on the onset and progression of AMD is controversial [26, 27]. In the current study, we did not detect altered serum levels of HDLC, lipoprotein a, cholesterol, and triglycerides. There was only a trend towards higher levels for apo B, which was reported previously by our group in a smaller case-control cohort [28]. Elevated levels of apo B lipoproteins are known to stimulate inflammation although the underlying aetiology of chronic subclinical inflammation is not clear [29].

Dietary assessments face the difficulties of variability in recall and reporting ability of subjects. Daily variability of human diet, use of different fats in cooking, differences in food preparation, consumption of processed food, or effects of food combinations present significant challenges. Our study was limited to the consumption of different food groups. No information was available on calorie intake, protein, carbohydrate, and fat levels of consumed food. We also did not evaluate dietary supplements or dietary patters. Our questionnaire did not include dairy products and had restricted power for fish and vegetable consumption due to limited distribution of answers.

However, our study has also several strengths. Multimodal imaging for AMD staging in EUGENDA avoided the misclassification of AMD phenotypes. Due to the complexity of the relationship between intake of various nutrients and environmental factors, we constructed a prediction model for AMD based on nutrition and adjusted for confounders. Addition of nutritional factors may thus further improve other prediction models for AMD based on demographic, genetic, and ocular factors.

In summary, we have identified different nutritional factors which may play a preventive role in AMD development. Further studies are needed to better understand the role of nutrition in the pathogenesis of AMD, also considering other genetic and environmental risk factors.

## Figures and Tables

**Table 1:** Demographic characteristics, median serum parameters, and nutritional factors.

	No AMD	Late AMD
Subjects	1773	1147
Age (mean) [years]∗	69.55 ± 7.55	77.06 ± 7.99
Age (range) [years]	51–100	53–98
BMI (mean) [kg/m^2^]∗	25.83 ± 3.88	26.10 ± 4.12
Male sex (%)	767 (43.3%)	467 (40.7%)
Serum lipids and lipoproteins		
Apolipoprotein B [mg/L]	965.15	1001.65
Apolipoprotein A2 [mg/L]	1557.90	1620.00
Lipoprotein a [U/L]	166.00	150.00
Cholesterol [mmol/L]	5.80	5.8
Triglycerides [mmol/L]	1.75	1.66
HDLC [mmol/L]	1.41	1.43
Fish intake		
<Once a week	407	247
Once a week	859	442
2–6x/week	434	206
Daily intake	9	4
No answer	64	248
Fruit intake		
<Once a week	54	54
Once a week	42	21
2–6x/week	276	132
Daily intake	341	694
No answer	60	246
Red meat intake		
<Once a week	395	157
Once a week	324	105
2–6x/week	852	527
Daily intake	127	113
No answer	75	245
Vegetable intake		
<Once a week	12	6
Once a week	12	5
2–6x/week	272	150
Daily intake	1418	739
No answer	59	247
Alcohol intake		
Irregular	706	432
Regular	1020	610
No answer	47	105
Smoking		
Never	965	623
Ever	768	415
No answer	40	109

BMI: body mass index; HDLC: high density lipoprotein cholesterol; data are median unless otherwise noted, ∗mean ± SD values.

**Table 2:** Univariate binary logistic regression analysis for late versus no AMD, adjusted for age and gender.

	OR	95% CI	*P* value
Body mass index (BMI) [kg/m^2^]			
Normal (BMI < 25)	1		
Overweight (BMI 25–29.99)	1.02	0.845–1.232	0.83
Obese (BMI ≥ 30)	1.46	1.121–1.899	0.005
Serum lipids and lipoproteins∗			
Apolipoprotein B	1.00	1.000–1.001	0.11
Apolipoprotein A2	1.00	1.000–1.001	0.31
Lipoprotein a	1.00	1.000–1.000	0.84
Cholesterol	1.05	0.943–1.165	0.38
Triglycerides	1.01	0.895–1.149	0.83
HDLC	1.04	0.722–1.496	0.84
Fish intake			
<Once a week	1		
Once a week	0.86	0.686–1.063	0.16
2–6x/week	0.79	0.614–1.018	0.07
Daily intake	0.45	0.114–1.804	0.26
Fruit intake			
<Once a week	1		
Once a week	0.60	0.295–1.197	0.15
2–6x/week	0.57	0.350–0.921	0.02
Daily intake	0.49	0.314–0.753	0.001
Red meat intake			
<Once a week	1		
Once a week	0.98	0.709–1.344	0.88
2–6x/week	1.77	1.393–2.248	2.94 × 10^−6^
Daily intake	2.39	1.679–3.397	1.27 × 10^−6^
Vegetable intake			
<Once a week	1		
Once a week	1.28	0.253–6.462	0.77
2–6x/week	2.17	0.681–6.899	0.19
Daily intake	1.85	0.593–5.797	0.29
Alcohol intake	1.11	0.932–1.327	0.24
Smoking	1.55	1.292–1.864	2.64 × 10^−6^

HDLC: high density lipoprotein cholesterol; OR: odds ratio; CI: confidence interval.

∗Reference: serum lipid and lipoprotein levels of control subjects.

**Table 3:** Multivariate binary logistic regression analysis for late versus no AMD.

	OR	95% CI	*P* value
Age	1.13	1.117–1.147	1.00 × 10^−13^
Gender			
Male	1		
Female	1.21	0.982–1.490	0.07
Body mass index (BMI) [kg/m^2^]			
Normal (BMI < 25)	1		
Overweight (BMI = 25–29.99)	0.99	0.806–1.226	0.96
Obese (BMI ≥ 30)	1.44	1.076–1.920	0.01
Fish intake			
<Once a week	1		
Once a week	0.95	0.748–1.195	0.64
2–6x/week	0.91	0.694–1.202	0.52
Daily intake	0.68	0.170–2.728	0.59
Fruit intake			
<Once a week	1		
Once a week	0.68	0.326–1.427	0.31
2–6x/week	0.58	0.348–0.961	0.04
Daily intake	0.52	0.328–0.823	0.005
Red meat intake			
<Once a week	1		
Once a week	1.04	0.746–1.448	0.82
2–6x/week	1.67	1.296–2.162	7.982 × 10^−5^
Daily intake	2.34	1.610–3.400	8.224 × 10^−6^
Alcohol intake	1.00	0.812–1.228	0.99
Smoking	1.56	1.268–1.922	2.743 × 10^−5^

Multivariate analysis included age, gender, body mass index, fish intake, fruit intake, red meat intake, alcohol intake, and smoking.

OR: odds ratio; CI: confidence interval.
