# Corrigendum to “Nutritional Risk Factors for Age-Related Macular Degeneration”

**DOI:** 10.1155/2016/7589328

**Published:** 2016-11-03

**Authors:** Lebriz Ersoy, Tina Ristau, Yara T. Lechanteur, Moritz Hahn, Carel B. Hoyng, Bernd Kirchhof, Anneke I. den Hollander, Sascha Fauser

**Affiliations:** ^1^Department of Ophthalmology, University Hospital of Cologne, Kerpener Straße 62, 50924 Cologne, Germany; ^2^Department of Ophthalmology, Radboud University Nijmegen Medical Center, Geert Grooteplein-Zuid 10, 6525 GA Nijmegen, Netherlands; ^3^Institute of Medical Statistics, Informatics and Epidemiology, University of Cologne, Kerpener Straße 62, 50924 Cologne, Germany

In the article titled “Nutritional Risk Factors for Age-Related Macular Degeneration” [1], “Apolipoprotein A2” should be replaced with “Apolipoprotein A1” throughout the text.

## References

[1] Ersoy L., Ristau T., Lechanteur Y. T. (2014). Nutritional risk factors for age-related macular degeneration. *BioMed Research International*.

